# Phospholipid Binding Protein C Inhibitor (PCI) Is Present on Microparticles Generated *In Vitro* and *In Vivo*

**DOI:** 10.1371/journal.pone.0143137

**Published:** 2015-11-18

**Authors:** Katrin Einfinger, Sigrun Badrnya, Margareta Furtmüller, Daniela Handschuh, Herbert Lindner, Margarethe Geiger

**Affiliations:** 1 Center of Physiology and Pharmacology, Department of Vascular Biology and Thrombosis Research, Medical University of Vienna, Vienna, Austria; 2 Center of Physiology and Pharmacology, Department of Physiology, Medical University of Vienna, Vienna, Austria; 3 Biocenter, Division of Clinical Biochemistry, Innsbruck Medical University, Innsbruck, Austria; National Cerebral and Cardiovascular Center, JAPAN

## Abstract

Protein C inhibitor is a secreted, non-specific serine protease inhibitor with broad protease reactivity. It binds glycosaminoglycans and anionic phospholipids, which can modulate its activity. Anionic phospholipids, such as phosphatidylserine are normally localized to the inner leaflet of the plasma membrane, but are exposed on activated and apoptotic cells and on plasma membrane-derived microparticles. In this report we show by flow cytometry that microparticles derived from cultured cells and activated platelets incorporated protein C inhibitor during membrane blebbing. Moreover, protein C inhibitor is present in/on microparticles circulating in normal human plasma as judged from Western blots, ELISAs, flow cytometry, and mass spectrometry. These plasma microparticles are mainly derived from megakaryocytes. They seem to be saturated with protein C inhibitor, since they do not bind added fluorescence-labeled protein C inhibitor. Heparin partially removed microparticle-bound protein C inhibitor, supporting our assumption that protein C inhibitor is bound via phospholipids. To assess the biological role of microparticle-bound protein C inhibitor we performed protease inhibition assays and co-precipitated putative binding partners on microparticles with anti-protein C inhibitor IgG. As judged from amidolytic assays microparticle-bound protein C inhibitor did not inhibit activated protein C or thrombin, nor did microparticles modulate the activity of exogenous protein C inhibitor. Among the proteins co-precipitating with protein C inhibitor, complement factors, especially complement factor 3, were most striking. Taken together, our data do not support a major role of microparticle-associated protein C inhibitor in coagulation, but rather suggest an interaction with proteins of the complement system present on these phospholipid vesicles.

## Introduction

Microparticles (MPs) are plasma membrane-derived vesicles with a size of 0.1–1 μm. In blood they can be released more or less by all cell types, e.g. platelets, erythrocytes, leukocytes, and endothelial cells, in response to cell activation or apoptosis [1, 2]. MP formation is preceded by an increase in intracellular calcium leading to a loss of membrane asymmetry with exposure of phosphatidylethanolamine (PE) and phosphatidylserine (PS). Degradation of the cytoskeleton and cellular contraction lead to blebbing of the plasma membrane with ultimate MP release [3–5]. MPs expose a panel of phospholipids and proteins on their surface, which are specific for the cellular origin and the type of stimulus that caused their release [6–8]. MPs in plasma reflect a dynamic balance between release and clearance from the circulation by phagocytosis [9–11].

In the plasma of healthy individuals about 70–90% of circulating MPs are considered to derive from platelets [12]. Recent studies demonstrated, however, that the majority of these MPs directly originate from megakaryocytes rather than from activated platelets [13, 14]. MP origin as well as their number and composition are altered in cardiovascular diseases [2, 15, 16]. MPs possess procoagulant and anticoagulant properties and may therefore contribute to thromboembolic processes. Surface-exposed phospholipids, in particular PS, as well as tissue factor promote coagulation [17–19]. Thrombomodulin, tissue factor pathway inhibitor, endothelial protein C receptor, and protein S were also detected on the MP surface, suggesting that they may also have anticoagulant properties [20–24].

Human protein C inhibitor (PCI, PAI3) is a secreted non-specific *ser*ine *p*rotease *in*hibitor (serpin) circulating in plasma at a concentration of 80–100 nM [25, 26]. In coagulation PCI has a dual role. By inhibiting activated protein C (aPC) as well as the activation of protein C by the thrombin/thrombomodulin complex, PCI promotes procoagulant processes [27–31]. Plasma PCI (pPCI) was shown to be the primary physiological inhibitor of aPC, and an increase in the level of aPC-PCI complexes is a highly sensitive parameter for ongoing coagulation in patients with intravascular coagulation [32, 33]. PCI also exhibits anticoagulant properties by directly inhibiting thrombin, factor Xa, factor XIa and plasma kallikrein [28, 34].

As a member of the serpin family of protease inhibitors PCI acts as a suicide substrate by forming a covalent complex with its target proteases [25, 35, 36]. The activity and target enzyme specificity of PCI can be modified by heparin and other glycosaminoglycans. Heparin stimulates the inhibition of aPC and thrombin by PCI, but interferes with the inhibition of tissue kallikrein by PCI [37–40]. Specific negatively charged or oxidized phospholipids such as PS, phosphoinositides, or oxidized PE bind to PCI. These phospholipids modulate its activity in a heparin-like manner [41–43]. PCI is secreted from α-granules of activated platelets and binds to platelet phospholipids. Synthetic phospholipid vesicles composed of 40% PE, 20% PS and 40% phosphatidylcholine (PC) significantly stimulated the inhibition of aPC by PCI. In these vesicles PE was identified as the critical phospholipid for increased PCI activity [42]. Furthermore, PCI can be internalized by cells in a process, which requires PE as well [44]. Immunohistochemical staining of atherosclerotic plaques suggested that co-localization of PCI with PS on apoptotic cells also occurs in an *in vivo* environment [41]. A recent study published by our group has shown that PCI inhibited phagocytosis of activated, PS-exposing platelets by human blood-derived monocytes [45].

PE and PS are normally localized to the inner leaflet of the plasma membrane bilayer, but are exposed on activated and apoptotic cells as well as on MPs. Binding of PCI to PS or PE on MPs may modulate PCI activity, thereby contributing to the procoagulant and/or anticoagulant properties of MPs. Furthermore, MPs may represent platforms for assembling PCI and its interaction partners. Therefore, this study was undertaken to analyze the occurrence of PCI in/on MPs isolated from different origins (cultured cells, activated platelets, human plasma). Having shown that these MPs contain PCI, we also determined its activity and possible binding partners in/on MPs derived from human plasma.

## Materials and Methods

The study was approved by the Ethics Committee of the Medical University of Vienna (approval number: EK Nr: 1448/2013). Written informed consent was obtained from all blood donors (10 males and 10 females, age 25–55 years).

### Cell culture and generation of cell-derived MPs

Human Jurkat T-lymphocytes and U937 myeloid cells (ATCC, American Type Culture Collection, Manassas, USA) were cultured in RPMI-1640 medium (Sigma-Aldrich, Austria) supplemented with 10% fetal bovine serum (FBS, Sigma-Aldrich, Austria), 1% penicillin/streptomycin/fungizone (PSF, Lonza, Austria) and 1% L-glutamine (Lonza, Austria). Cells were maintained in a humidified incubator with 5% CO_2_ at 37°C as published earlier [45]. Cells were harvested by centrifugation (500xg, 5 min, 20°C). The resulting pellet was washed twice with serum-free medium and resuspended in serum-free medium at a concentration of 1x10^7^ cells/ml. The cell suspension was seeded in wells (3 ml each) of a 6-well plate (Iwaki, Japan). To induce apoptosis and MP release, cells were either treated with staurosporine (STS, 1 μM final concentration (fc), Sigma Aldrich, Austria) or kept in serum-free medium (serum starvation, control). After 3, 5.5 and 18 hours cells were centrifuged at 500xg for 5 min at 20°C. Conditioned media were aspirated and centrifuged (1,500xg, 10 min, 20°C) to remove cell debris. Resulting supernatants, containing MPs, were carefully aspirated and stored in aliquots (750 μL each) at -70°C until analyses. Cells (5x10^5^/tube) were stained with annexin V-EGFP and propidium iodide (PI) according to the manufacturer’s protocol (apoptosis kit, BioVision, USA) and analyzed by flow cytometry (FACS Calibur, Becton Dickinson, USA). For each sample 5,000 particles were measured. Data were analyzed using WinMDI 2.8 Software (freeware, developer: Joe Trotter).

### Blood samples and preparation of platelet-free plasma (PFP)

For preparation of platelets blood from 4 female healthy donors (age 26–33 years) was obtained. Venous blood was collected in Vacuette tubes containing 3.8% sodium citrate (Greiner Bio-one GmbH, Austria) using a 21-gauge needle (Becton Dickinson Austria GmbH, Austria) without applying venostasis. The first 3 ml of blood were discarded. Platelet-poor plasma was prepared by centrifugation at 1,500xg for 10 min at 20°C. Aspirated supernatants were centrifuged again to obtain PFP (1,500xg, 10 min, 20°C) containing MPs. This PFP from individual donors was stored in aliquots (250 μl each) at -70°C [46].

### MP generation from isolated platelets

Blood was collected from 4 female healthy donors (age 26–33 years) as described above, and platelets were isolated as previously described [47]. In brief, citrated blood was centrifuged at 120xg for 20 min at 20°C to obtain platelet-rich plasma (PRP). Platelets were isolated from PRP by centrifugation at 3,000xg for 2 min at 20°C in the presence of prostacyclin (PGI_2_, 1 μM, Sigma-Aldrich, Austria), followed by repeated washing in sterile filtered (0.1 μm) phosphate-buffered saline (PBS: 133 mM NaCl, 1.3 mM KCl, 2.5 mM Na_2_HPO_4_x2H_2_O, 0.5 mM KH_2_PO_4_, pH = 7.4).

MP generation was induced by addition of thrombin receptor activating peptide-6 (TRAP–6, 20 μM, AnaSpec, Belgium) or ionomycin (40 μM, Santa Cruz Biotechnology, Inc., Germany) in the presence and absence of pPCI (300 nM) to isolated platelets (300,000/μl) in PBS. After 20 min of stimulation platelets were centrifuged at 3,000xg for 5 min to obtain MP containing platelet-free supernatant.

### Isolation of MPs from conditioned media and PFP

Isolation of MPs was performed according to a slightly modified method as published elsewhere [46]. Briefly, frozen conditioned media (750 μl portions) or PFP (250 μl portions) were slowly thawed on melting ice. MPs were pelleted by centrifugation at 18,000xg for 30 min at 4°C. Supernatants were removed, and MPs were washed with PBS containing 0.38% Na-citrate and centrifuged again. Depending on the subsequent assay, MPs were washed 1 to 3 times with PBS-citrate. All buffers were filtered (0.22 μm).

### Enzyme linked immunosorbent assay (ELISA)

Microtiter plates (Nunc maxisorp, Nunc, Denmark) were coated with annexin V (50 μL/well, Sigma-Aldrich, Austria) at a concentration of 10 mg/l in Tris-buffered saline (TBS: 50 mM Tris-HCl, 0.1 M NaCl, pH = 7.4) overnight at 4°C. Wells were blocked with 50 μL bovine serum albumin (BSA, Biomol, Germany, 10 g/l) for 60 min at room temperature (RT) and then washed 3 times with TBS containing 0.1% Tween 20 [48]. MPs were isolated from PFP (25, 50, 100, 200 μl) as described above, washed 3 times with PBS-citrate, resuspended each in 50 μl annexin V binding buffer (apoptosis kit, Biovision, USA), added to the wells and incubated for 1 hour at 37°C. After washing with TBS containing 0.1% Tween 20 (3 times), wells were incubated with polyclonal rabbit anti-PCI-IgG (7.4 μg/ml) in TBS-1% BSA for 1 hour at 37°C. Wells were washed prior to the addition of peroxidase-coupled donkey anti-rabbit-IgG (0.11 μg/ml, GE Healthcare, Austria) in TBS-1% BSA (1 hour, 37°C). After washing, the signal was detected with tetramethylbenzidine (TMB) solution (VWR, Austria). Reactions were stopped after 20 min at RT with 50 μL of 1 M H_3_PO_4_. The absorbance was measured at 450 nm with a Synergy H4 microplate reader (BioTek, USA).

### Labeling of anti-PCI-IgG and plasma PCI (pPCI)

Monoclonal mouse anti-PCI-IgG (4PCI, Technoclone, Austria) [37] was conjugated with Alexa Fluor 633 (AF633 labeling kit, Molecular Probes, Austria) according to the instructions of the manufacturer. pPCI purified as described earlier [37] was dialyzed against PBS and conjugated with Cy3 (Cy3 labeling kit, Amersham Biosciences Europe GmbH, Germany).

### Labeling of MPs for flow cytometry

For each reaction MPs contained in 100 μl PFP or 750 μl cell supernatants were used. MPs were washed once with PBS-citrate, blocked with PBS-4% BSA (fc) and incubated with anti-PCI-IgG-AF633 (50 μg/ml), anti-CD41-IgG-PE (3 μg/ml, BioCytex, France), anti-CD62P-IgG-PE (P-selectin, 0.625 μg/ml, BD Bioscience, Austria), and annexin V-EGFP (30 μg/ml, BioVision, USA) in 50 μl PBS or 500 μl annexin V binding buffer. MPs were incubated successively with each antibody for 30 min on ice in PBS (antibodies) or annexin V binding buffer, respectively. After each incubation step MPs were washed once with PBS and centrifuged. Concentration-matched isotype antibodies (IgG1-PE/clone 2DNP-2H11, IgG-AF633) or annexin V-EGFP binding in calcium-free buffer were used as controls. MPs were fixed with paraformaldehyde (1% fc) for 20 min at RT and analyzed by flow cytometry.

Platelet-derived MPs were labeled with anti-PCI-IgG-AF633, annexin V-EGFP, and anti-CD62P-IgG-PE and analyzed in annexin V binding buffer immediately without fixation.

### Binding of pPCI-Cy3 to MPs

For each reaction MPs contained in 100 μl PFP were used. MPs were washed once with PBS-citrate and incubated with pPCI-Cy3 (100 and 500 nM) for 1 hour at RT in 100 μl PBS. MPs were washed with PBS, stained with annexin V-EGFP as indicated earlier and subjected to flow cytometry. Apoptotic Jurkat cells [45] were incubated with 100 nM pPCI-Cy3 in parallel to MPs. *In vitro* generated MPs derived from Jurkat cells upon serum starvation or STS treatment (1 μM, 18 hours, 37°C) were incubated with pPCI-Cy3 (80 nM) in the absence or presence of heparin (10 U/ml) in PBS for 45 min at RT.

### Flow Cytometry of MPs

Analyses were performed on a FACSCanto II flow cytometer (BD Bioscience, Austria). The set-up of the flow cytometer was performed according to a slightly modified protocol described elsewhere [49]. Standardization of MP analyses was achieved using a fluorescent bead mixture (0.5, 0.9, 3 μm, Megamix, BioCytex, France). Forward scatter (FS), side scatter (SS) and all fluorescent channels were plotted on logarithmic scales. The upper limit of the MP region was defined by the end of the 0.9 μm bead cloud. Annexin V labeling was used to distinguish MPs from background noise. For each sample 10,000 particles were measured. Data were analyzed using FCS Express V3 (DeNovo, USA).

### Inhibition of aPC or thrombin by MPs

Assays were performed in 96-well microtiter plates (Greiner Bio-One, Austria). For each reaction a MP suspension containing a number of MPs equivalent to that present in 800 μl PFP was used. MPs were washed 3 times with PBS-citrate to thoroughly remove pPCI. aPC (1 nM, Baxter, Austria) or thrombin (1 nM, Technoclone, Austria) were incubated with MPs in the absence or presence of unfractionated heparin (10 U/ml fc) in 100 μl Hepes-buffered saline (25 mM Hepes, 137 mM NaCl, 3.5 mM KCl, 3 mM CaCl_2_.H_2_O, pH = 7.4) or TBS (10 mM Tris, 100 mM NaCl, pH = 7.4), respectively. Each buffer contained 1% BSA. In some experiments recombinant human (rh)PCI (10 nM and 50 nM) was added. rhPCI was prepared as described earlier [50]. Mixtures were incubated for 20 min at 37°C. Thereafter, 100 μl of the chromogenic substrates S-2366 for aPC or S-2238 for thrombin (0.4 mM fc, Chromogenix, Italy) dissolved in Hepes or TBS were added to each well, respectively. The colour development was determined with a Synergy H4 microplate reader at 405 nm. The remaining aPC or thrombin activity without MPs, PCI and heparin was assigned to 1, and the aPC or thrombin activity was calculated for each reaction mixture.

### Incubation of MPs with heparin

For each reaction MPs contained in 100 μl PFP were washed once with PBS-citrate and incubated without or with different concentrations of unfractionated heparin (0.05–50 U/ml, Baxter, Austria) for 30 min at RT in PBS. After washing with PBS, MPs were stained with anti-PCI-IgG-AF633 and annexin V-EGFP as stated above and analyzed by flow cytometry. For Western blotting MPs (each reaction containing a number of MPs equivalent to that present in 2 ml PFP) were washed 3 times with PBS-citrate, incubated with unfractionated heparin (50 U/ml) for 0, 15, 30 and 45 min in 50 μl PBS and thereafter pelleted by centrifugation (18,000xg for 30 min at 4°C). Aspirated supernatants (30 μl) were added to an equal volume of Laemmli buffer, and samples were heated for 10 min at 95°C and stored at -70°C.

### Immunoprecipitation of PCI from MPs

MPs were isolated from 16 ml PFP, washed 3 times with PBS-citrate, and lysed with 62 nM n-octyl-β-D-glycopyranoside (Sigma Aldrich, Austria) and 10 nM ethylenediaminetetraacetate (Sigma Aldrich, Austria) containing a protease inhibitor cocktail (Complete, Roche, Austria) (pH = 7.4) for 15 min at 37°C on a shaker. The MP lysate was dialyzed against 10 mM Tris, 500 mM NaCl (pH = 7.4) overnight at 4°C and thereafter incubated with polyclonal rabbit anti-PCI-IgG coupled to Sepharose (GE Healthcare, Austria) for 3 hours at 4°C on a shaker. Sepharose beads were washed 5 times with dialysis buffer (10,000xg, 30 sec, 4°C). Bound proteins were eluted from the Sepharose beads with 80 μl of Laemmli buffer (125 mM Tris, 20% glycerol, 4.1% SDS, 0.01% bromphenol blue) prediluted with an equal volume of PBS. Samples were heated at 95°C for 10 min, centrifuged and supernatants were stored at -70°C.

### SDS PAGE, Western Blotting and Page Blue Staining

SDS PAGE was performed with 10% acrylamide gels according to the method of Laemmli [51] and followed by Western blotting. After protein transfer, PVDF membranes were blocked with PBST (135 nM NaCl, 1.3 mM KCl, 2.5 mM Na_2_HPO_4_, 0.5 mM KH_2_PO_4_, 0.1% Tween-20) containing 5% dry milk (Sigma-Aldrich, Austria) for 1 hour at RT. Primary antibodies were incubated in PBST-5% milk at 4°C overnight. Membranes were washed 5 times for 5 min each in PBST. The secondary peroxidase-labeled antibody was applied for 45 min in PBST-5% milk. After washing, peroxidase reactions were detected with SuperSignal West Femto (Thermo Scientific, Austria). Primary antibodies used were monoclonal mouse anti-serpinA5-IgG (0.5 μg/ml, R&D Systems, UK) and polyclonal rabbit anti-PCI-IgG (7.4 μg/ml) [37]. The respective secondary antibodies were peroxidase-conjugated donkey anti-rabbit-IgG (0.11 μg/ml, GE Healthcare, Austria), and HRP-conjugated sheep anti-mouse-IgG (0.13 μg/ml, GE Healthcare, Austria). Page Blue staining of SDS-gels was performed according to the product manual (PageBlue Protein staining solution, Thermo Fisher Scientific Biosciences GmbH, Austria).

### Mass spectrometry analysis of the protein digests

The in-gel digestion was performed as published previously [52]. Protein digests were analyzed using an UltiMate 3000 nano-HPLC system (Dionex, Germany) connected online to a linear ion-trap mass spectrometer (LTQ Velos; ThermoScientific) equipped with a nanospray ionization source. The method applied was recently described by Sarg et al. [53]

### Database search

MS/MS spectra were searched against a NCBI nr human protein database via SEQUEST, Proteome Discoverer software (Version 1.2, ThermoScientific). Database search criteria were set as follows: Processing MS^n^: allowed cleavage sites, Lys and Arg; maximum miss cleavage sites, 2; precursor tolerance 0.5 Da; fragment mass tolerance, 0.8 Da. The variable modifications considered were carbamidomethylation (+57.021) at Cys and oxidation (+15.995) at Met. All data were filtered to satisfy a false discovery rate (FDR) of 1% or better.

### Statistical Analyses

Statistical significance of differences was calculated in GraphPad PRISM Software (GraphPad Software Inc., USA) using One-way ANOVA and the Bonferroni’s multiple comparison test. *p* < 0.05 was considered as significant.

## Results

### MPs derived from cultured cells and platelets expose PCI on their surface

**Fig 1:**
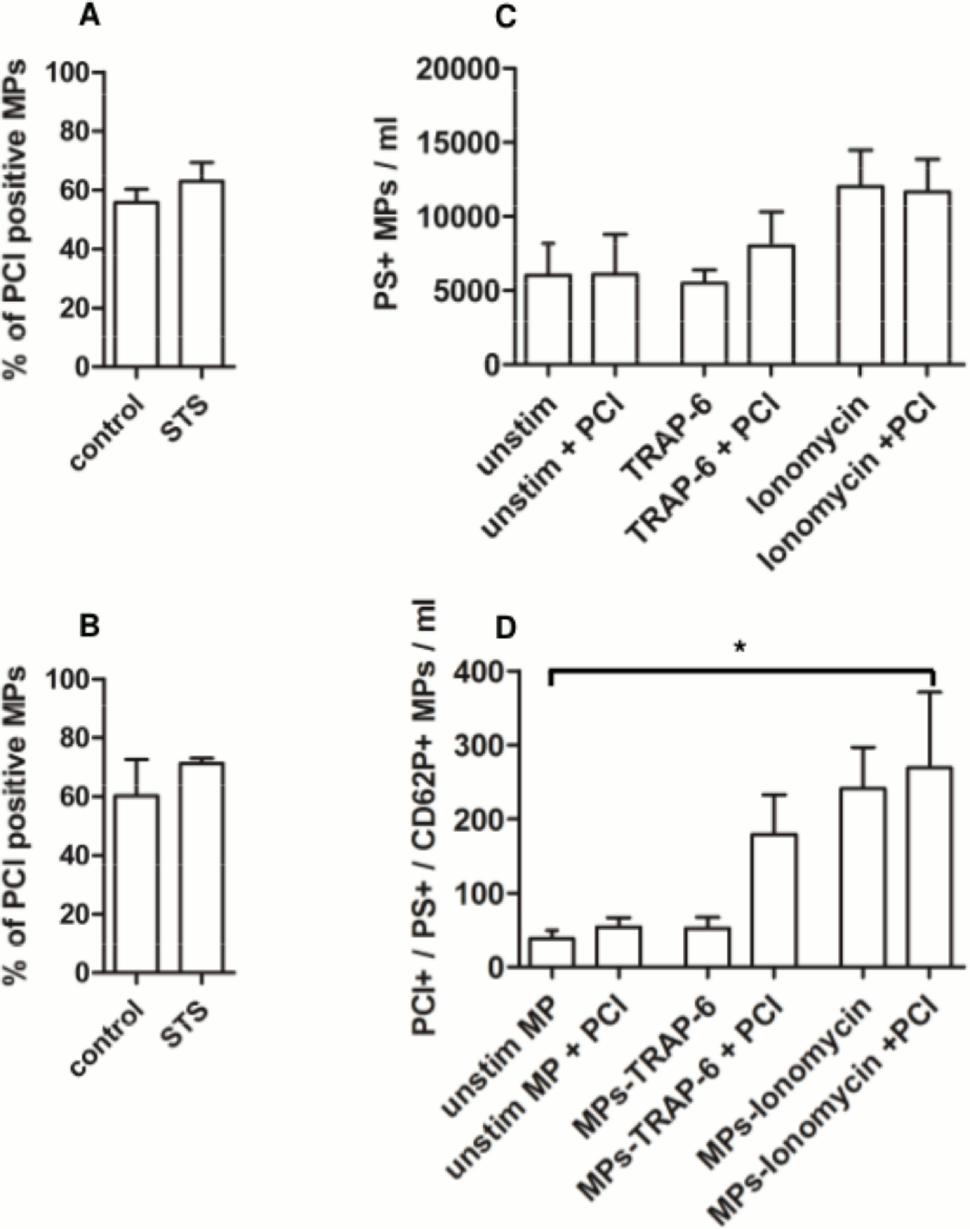
MPs derived from cultured cells and platelets expose PCI on their surface. Jurkat cells and U937 cells were cultured in serum-free media (serum starvation, control) or treated with STS to induce apoptosis. MPs, isolated from the conditioned media of (A) Jurkat cells and (B) U937 cells, were stained with annexin V-EGFP and anti-PCI-IgG-AF633 and subjected to flow cytometry. MPs were defined as events with a size smaller than 1 μm, which show binding of annexin V. Data shown represent means and range of two independent experiments performed in duplicates. Washed platelets (n = 4) were activated with TRAP-6 (20 μM) or ionomycin (40 μM) to induce membrane blebbing and MP release. MP levels obtained from resting platelets served as control. MPs were analyzed by flow cytometry after staining with annexin V-EGFP, anti-PCI-IgG-AF633 and anti-CD62P-IgG-PE. (C) Stimulus dependent numbers of MPs (annexin V positive events). (D) Exposure of PCI on PS/CD62P double positive MPs after platelet activation with TRAP-6 (20 μM) or ionomycin (40 μM) in the absence or presence of pPCI (300 nM).

In order to analyze whether endogenous PCI is contained in/on MPs released during membrane blebbing, Jurkat cells and U937 cells were treated with STS to induce apoptosis. STS-treated and control cells were kept in serum-free media to exclude binding of PCI present in FBS. After 18 hours, cells were harvested and the presence of apoptotic cells was determined by flow cytometry after staining with annexin V-EGFP and PI (see S1 Fig). MPs were isolated from the supernatants by differential centrifugation, stained with annexin V-EGFP and anti-PCI-IgG-AF633 and subjected to flow cytometry. MPs were defined as events smaller than 1 μm in size, which show annexin V binding. Between 55.8–71.3% of MPs generated from Jurkat cells (Fig 1A) and U937 cells (Fig 1B) exposed PCI on their surface. There was no statistically significant difference between MPs derived from STS-treated cells and control cells. These results indicate that endogenous PCI is incorporated into the plasma membrane of MPs during the process of membrane blebbing.

Since PCI is stored in α-granules of platelets [54] and released upon activation [42] we determined whether platelet activation results in the release of MPs exposing PCI on their surface. Therefore, washed platelets from healthy donors (n = 4) were activated with TRAP-6 (20 μM) or with ionomycin (40 μM) for 20 min. To rule out an influence of PCI on the release of MPs, the experiments were performed in the absence and presence of pPCI (300 nM). As depicted in Fig 1C, activation of platelets with TRAP-6 did not cause an increase in the number of annexin V positive MPs. However, when platelets were activated with ionomycin, the number of annexin V positive MPs increased 2-fold as compared to MPs released by unstimulated platelets reflecting the basal level of MP release. Co-incubation of platelets with pPCI and TRAP-6 or ionomycin did not alter the number of MPs released by platelets compared to the respective control without pPCI.

Furthermore, flow cytometry analyses revealed the presence of PCI on the surface of those MPs, which also expose PS and CD62P (Fig 1D). CD62P, an adhesion receptor stored within α-granules in resting platelets, is released upon activation and can subsequently be detected on the surface of activated platelets as well as on MPs. The number of MPs exposing PS, CD62P, and PCI did not increase upon platelet activation with TRAP-6. However, when exogenously added pPCI was present during platelet activation with TRAP-6, the number of annexin V positive, CD62P positive and PCI positive MPs increased >4-fold, suggesting that during or after MP formation exogenous pPCI is bound to their surface. In contrast, upon activation of platelets with ionomycin, MPs exposed a higher amount of PCI as compared to MP derived from unstimulated or TRAP-6 activated platelets, but the presence of pPCI during platelet activation did not result in additional binding of pPCI.

### Plasma-derived MPs from healthy donors expose PCI, PS and CD41 on their surface

**Fig 2:**
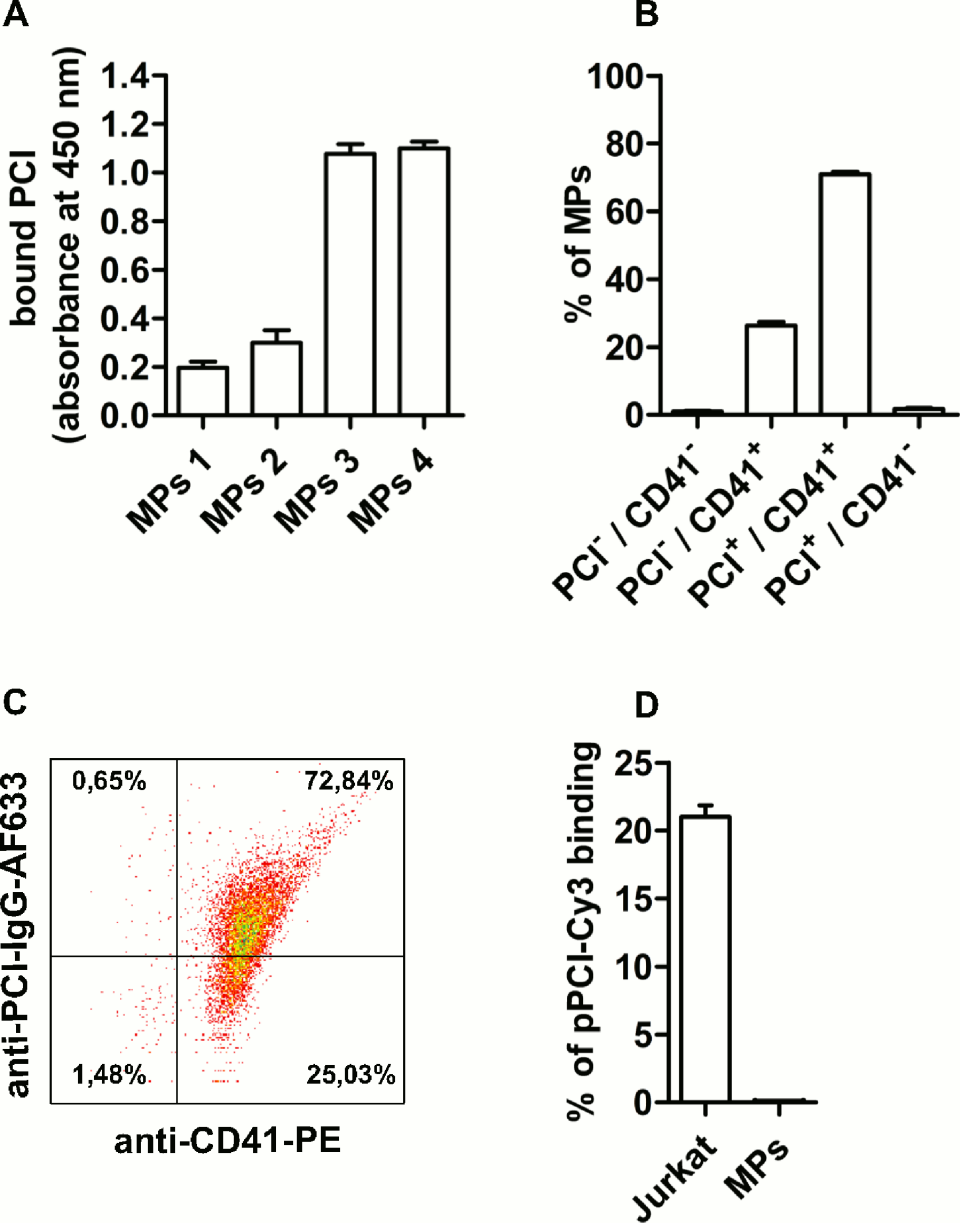
Plasma-derived MPs from healthy donors expose PCI, PS and CD41 on their surface and are saturated with pPCI. (A) Annexin V was immobilized on microtiter plates and incubated with different dilutions of MP suspensions obtained from pooled human plasma (MPs 1: 25 μl, MPs 2: 50 μl, MPs 3: 100 μl, MPs 4: 200 μl, n = 4). MP-bound PCI was detected with a rabbit anti-PCI-IgG. Absorbance values shown are corrected for non-specific binding of antibodies to annexin V and represent means and range of duplicates. (B) MPs isolated from plasma of individual healthy donors (n = 6) were stained with annexin V-EGFP, anti-PCI-IgG-AF633 and anti-CD41-IgG-PE (platelet/megakaryocyte specific marker) and subjected to flow cytometry. Data represent means and SEM, each performed in duplicates. (C) Representative dot plot of annexin V positive MPs showing bound PCI and CD41. (D) Binding of pPCI-Cy3 to the surface of MPs and Jurkat cells was assessed by flow cytometry. Plasma-derived MPs were incubated with pPCI-Cy3 (100 nM) for 1 hour at RT. Apoptotic Jurkat cells were used as positive control. Data represent means and range of two independent experiments (Jurkat) or means + SEM of three independent experiments (MPs), each performed in duplicates.

To determine if PCI is also present on *in vivo* formed MPs, we isolated MPs from plasma of individual healthy volunteers by differential centrifugation and studied the presence of PCI on these MPs. In all tested samples PS positive/PCI positive microparticles were detected by ELISA (n = 4, Fig 2A). Furthermore, up to 98% of annexin V positive MPs were CD41 positive and up to 73% of these CD41 positive MPs carried PCI, as determined by flow cytometry (n = 6, Fig 2B and 2C). To differentiate whether MPs were derived from megakaryocytes (CD62P negative) or from activated platelets (CD62P positive), flow cytometric analyses of MPs using CD62P as a platelet activation marker were performed. However, MPs did not show any CD62P positivity (data not shown), suggesting that they are primarily derived from megakaryocytes.

To quantify the amount of PCI on MPs we performed experiments with SDS PAGE and Western blotting and calculated the fraction of MP-associated PCI in the plasma of healthy individuals being 2 nM corresponding to 2% of the pPCI concentration (data not shown).

To investigate whether MPs bind exogenously added pPCI, MPs were incubated with different concentrations of pPCI-Cy3 (100 nM and 500 nM) for 1 hour at RT. Labelled pPCI was used to reliably distinguish binding of exogenous pPCI from already MP-bound PCI. Plasma-derived MPs of healthy individuals did not bind any exogenously added pPCI-Cy3 as determined by flow cytometry. As positive control we studied the binding of pPCI-Cy3 to apoptotic Jurkat cells known to bind pPCI-Cy3 [45]. In contrast to MPs from healthy individuals, about 20% of apoptotic Jurkat cells bound pPCI-Cy3 (Fig 2D). Also MPs derived from these cells bound pPCI-Cy3 (S2 Fig). This binding was independent on the method used to generate these MPs. Heparin inhibited binding of pPCI-Cy3 to Jurkat-derived MPs, when they were obtained by STS-treatment of the cells, but not (or much less), when they were generated by serum starvation.

### Heparin removes PCI from MPs

**Fig 3:**
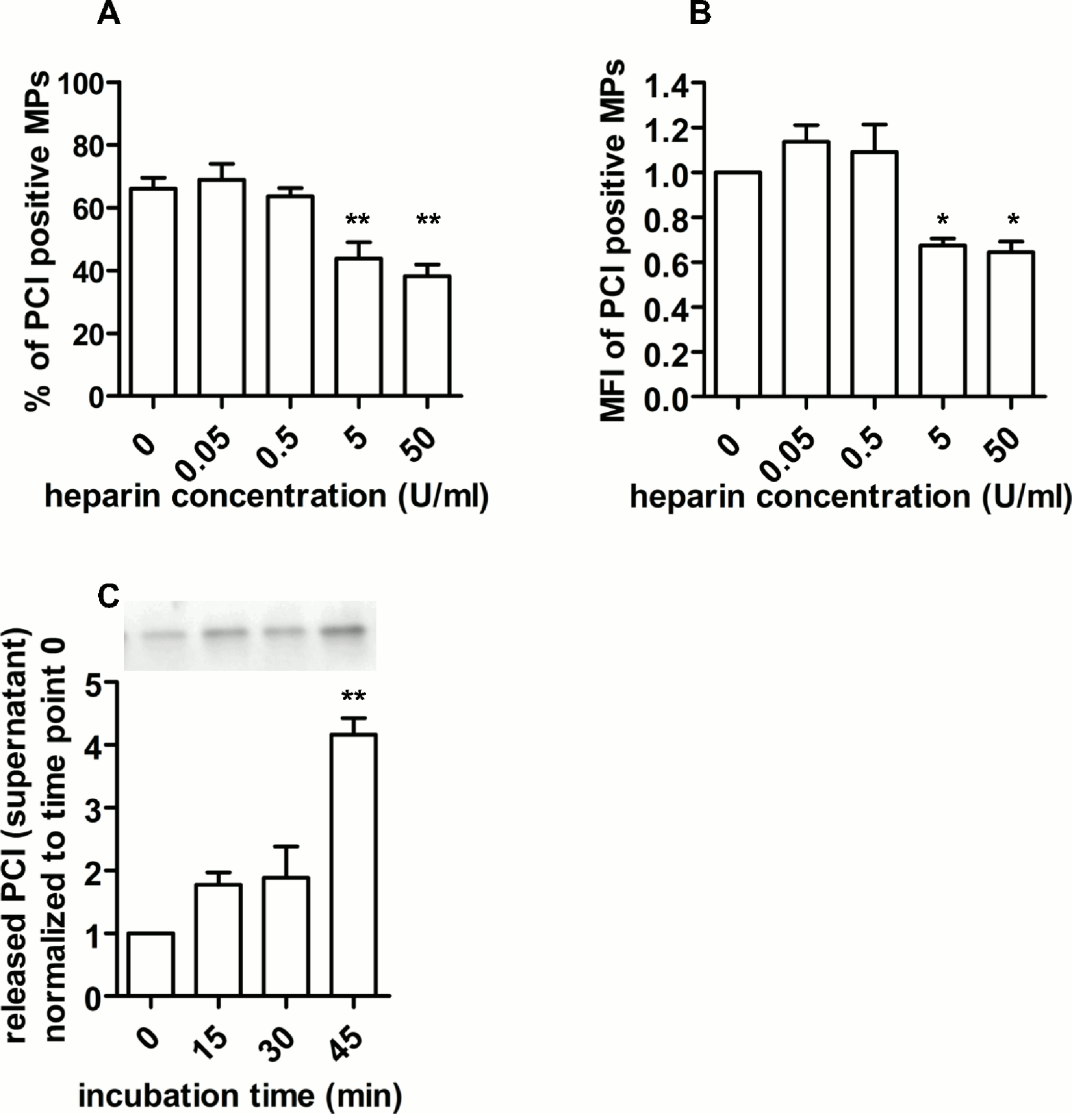
Heparin removes PCI from MPs. Plasma-derived MPs from healthy donors were incubated without or with different heparin concentrations (0.05, 0.5, 5, 50 U/ml) for 30 min (panels A and B) at RT. Surface-bound PCI was analyzed by flow cytometry after staining with anti-PCI-IgG-AF633 and annexin V-EGFP. (A) % of PCI positive MPs and (B) MFI of PCI positive MPs. Data represent means + SEM of four independent experiments performed with MPs isolated from pooled plasma (equal aliquots from 4 healthy individuals), each performed in duplicates. (C) Western blot probed with rabbit anti-PCI-IgG showing supernatants of MPs after incubation with heparin (50 U/ml) for the time periods indicated. PCI in supernatants was quantified and normalised to supernatants with heparin at time point 0. Data represent means and range of two independent experiments performed with MPs isolated from pooled plasma (equal aliquots from 4 healthy individuals).

To analyze whether heparin removes PCI from the surface of MPs, we determined the remaining MP-bound PCI after incubation with increasing heparin concentrations (0, 0.05, 0.5, 5, 50 U/ml, 30 min, RT) by flow cytometry. We could show that heparin removed surface-bound PCI from plasma-derived MPs in a concentration- and time-dependent manner (Fig 3). The percentage of PCI positive MPs was significantly lower after incubation with heparin at concentrations of 5 and 50 U/ml (Fig 3A) as was the geometric mean fluorescent intensity (MFI) showing that 33–36% of the MP-bound PCI was removed from the MP surface (Fig 3B). The time-dependent release of PCI by heparin is shown in Fig 3C. An incubation time of 30 min with heparin (50 U/ml) doubled the PCI concentration in the supernatant as a result of PCI release from the MP surface. PCI in the supernatant further increased up to 4-fold when MPs were incubated with heparin (50 U/ml) for 45 min as shown by SDS PAGE and Western blotting (Fig 3C). In the absence of heparin the release of PCI was very low and no clear time-dependence could be determined [see also Fig 2B (0 min incubation) and Fig 3A (30 min incubation)].

### MP-bound PCI does not inhibit aPC or thrombin. MPs do not modulate the activity of exogenously added rhPCI towards aPC or thrombin

**Fig 4:**
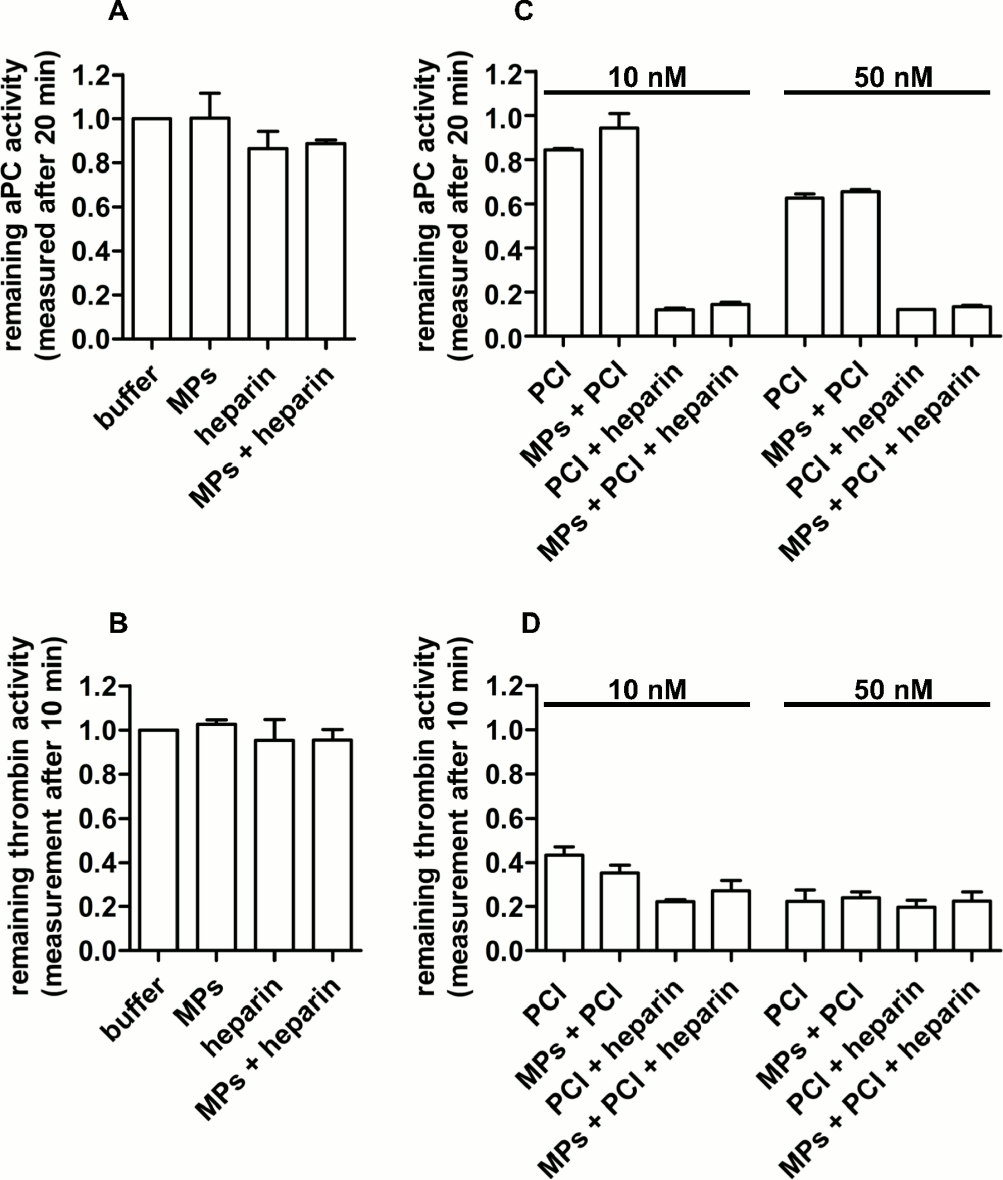
MP-bound PCI does not inhibit aPC or thrombin. MPs do not modulate the activity of exogenously added PCI. (A) aPC (1 nM) or (B) thrombin (1 nM) were incubated with plasma-derived MPs in the absence or presence of heparin (10 U/ml) and the remaining aPC or thrombin activity was determined. The effect of MPs on the inhibitory activity of exogenously added rhPCI (10 nM and 50 nM) towards (C) aPC and (D) thrombin was measured in the absence or presence of heparin (10 U/ml). Data represent means and range of two independent experiments performed with MPs isolated from pooled plasma (equal aliquots from 4 healthy individuals), each performed in duplicates.

Having shown the presence of PCI on the surface of MPs and its release by heparin, we determined its activity towards aPC and thrombin. As revealed by inhibition assays, MP-bound PCI did not inhibit aPC or thrombin, neither in the absence nor in the presence of heparin (10 U/ml) (Fig 4A and 4B). In these assays the concentration of MP-bound PCI was approximately 10 nM as judged from Western blotting of MP lysates. This concentration is high enough to inhibit aPC and thrombin (see Fig 4C and 4D). Even at higher MP concentrations per reaction corresponding to estimated 30 nM MP-bound PCI, we could not observe any inhibition of aPC (data not shown).

Although MPs expose PS as judged from annexin V binding, they did not stimulate the activity of exogenously added rhPCI. The inhibition of aPC and thrombin by rhPCI was comparable to the inhibition by rhPCI without MPs. However, in controls the addition of heparin (10 U/ml) stimulated the activity of rhPCI towards aPC and thrombin (Fig 4C and 4D).

### PCI in MP lysates co-precipitates with complement factors

**Fig 5:**
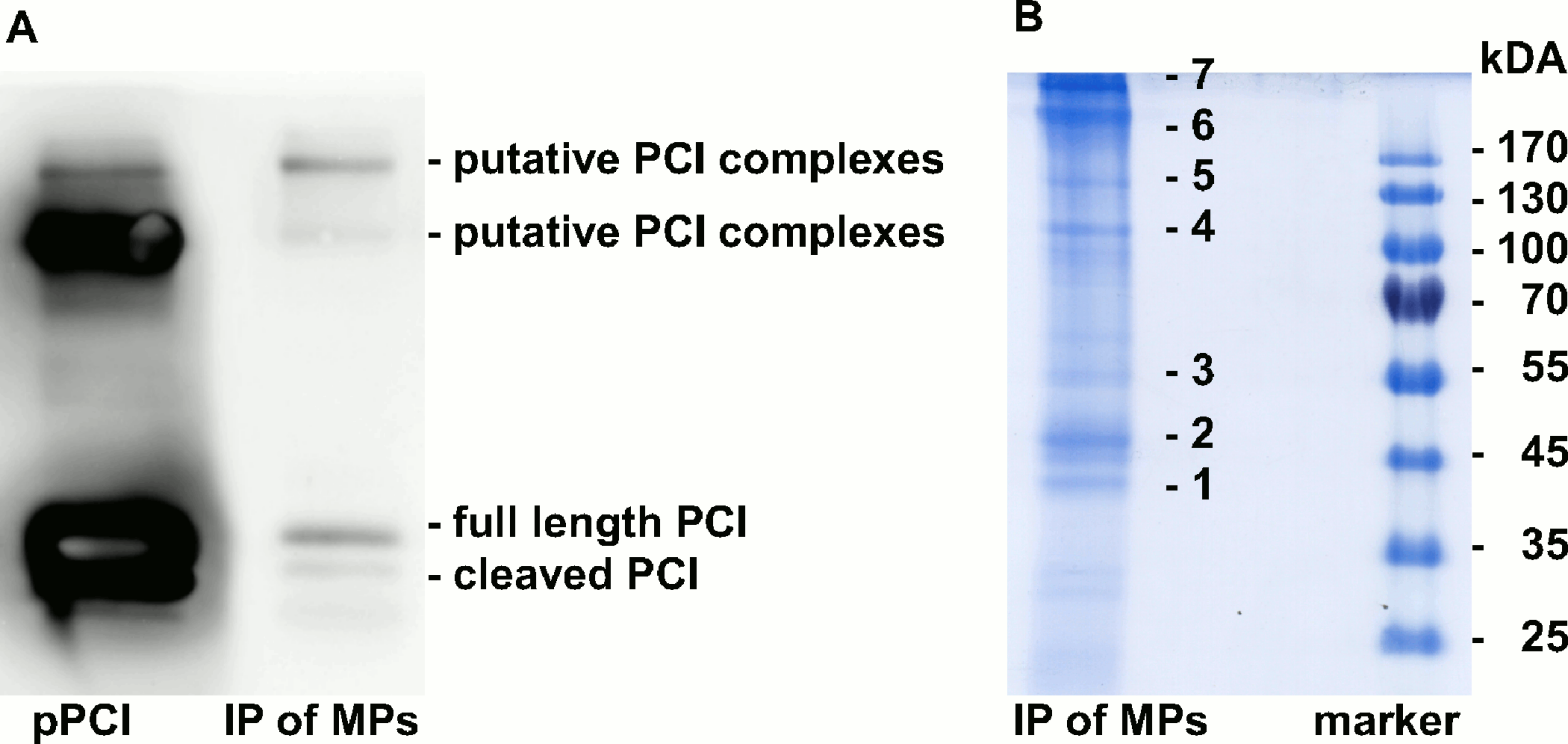
PCI in MP lysates co-precipitates with complement factors. Solubilized proteins contained in MP-lysates isolated from pooled plasma (16 ml) of healthy donors (n = 3) were immunoprecipitated with polyclonal rabbit-anti-PCI-IgG coupled to sepharose. Bound proteins were eluted with 80 μl of Laemmli buffer. (A) 18 μl of the immunoprecipitated MPs and pPCI (360 ng/lane) as control were loaded and run at a constant current of 120 mA for 2 hours. Proteins were transferred to a PVDF membrane and analyzed by Western blotting using monoclonal mouse-anti-PCI-IgG (left lane: pPCI, right lane: immunoprecipitate (IP) of MPs). (B) SDS PAGE with IP of MPs (18 μl/lane) followed by Page Blue staining (left lane: IP of MPs, right lane: molecular weight marker). Bands 1–7 (left lane) were further analyzed by mass spectrometry (results shown in Table 1).

In order to analyze the presence of binding partners of PCI in/on MP, solubilised proteins in MP lysates were immunoprecipitated with polyclonal rabbit-anti-PCI-IgG, subjected to SDS PAGE, and analyzed by Western blotting using monoclonal mouse-anti-PCI-IgG (Fig 5A). Purified pPCI was used as control, which exhibited the typical pattern of closely spaced bands corresponding to full length PCI (57 kDa) and reactive site cleaved PCI (54 kDa). For pPCI we detected also some characteristic high molecular weight bands corresponding to PCI complexes. In MP lysates we observed a band, which migrated with the same mobility as intact PCI (57 kDa), while a band corresponding to cleaved PCI (54 kDa) was hardly seen. We furthermore observed a high molecular weight band, suggesting the presence of PCI complexes in MP lysates.

SDS-PAGE gels were stained with Page Blue, and bands 1 to 7 (Fig 5B) were analyzed by mass spectrometry. PCI was detected in band 3. The presence of PCI complexes as suggested by the high molecular weight band seen in Western blots (Fig 5A) could be confirmed in band 7 by setting the FDR rate to less than 1% and requiring at least 2 peptides per protein. No protease was detectable in this band. The most prominent group of proteins co-precipitating with PCI were components of the complement system (Table 1).

**Table 1:** Complement factors identified after immunoprecipitation of PCI in MP lysates. Protein digests were analyzed by mass spectrometry and MS/MS spectra were searched against a NCBI nr human protein database via SEQUEST.

Band	Protein name	Accession number
1	complement component 3	gi119589476
2	complement component 3 precursor	gi115298678
	Vitronectin	gi13477169
3	Vitronectin	gi13477169
	C1q B-chain precursor	gi573114
	complement component 3 precursor	gi115298678
	complement C1q subcomponent subunit A precursor	gi7705753
4	complement component 3 precursor	gi115298678
	Vitronectin	gi13477169
	C1q B-chain precursor	gi573114
5	complement component 3 precursor	gi115298678
	Vitronectin	gi13477169
	C1q B-chain precursor	gi573114
	complement component 4a	gi179674
6	complement component 3 precursor	gi115298678
	C1q B-chain precursor	gi573114
7	complement component 3 precursor	gi115298678
	complement component 4a	gi443671
	C1q B-chain precursor	gi573114
	complement component 9	gi2258128

## Discussion

PCI is a heparin- and phospholipid-binding serpin, which binds PS, oxidized PE, PIPs, and cardiolipin [41, 43]. *In vivo* PCI may therefore be bound not only to glycosaminoglycans [55, 56], but also to negatively charged phospholipids such as exposed on apoptotic and activated cells or on MPs. *In vitro* these phospholipids modulate PCI activity in a heparin-like manner. Binding of PCI to anionic phospholipids exposed on cells or MPs may therefore regulate PCI activity *in vivo*. The present study was undertaken to analyze the occurrence of PCI on MPs derived from cultured cells, activated platelets and from plasma of healthy individuals. Having shown the presence of PCI on MPs from different origin, we further set about to determine the activity of PCI as well as its binding partners in/on plasma-derived MPs from healthy individuals.

A number of agonists can be used to generate MPs *in vitro*. We used STS, which–according to the literature–releases the highest number of MPs from Jurkat cells [57]. To determine whether PCI is incorporated into MPs during membrane blebbing, we used Jurkat cells and U937 cells, which contain an endogenous pool of PCI. We can show for the first time that MPs derived from Jurkat cells and U937 cells incorporated PCI during membrane blebbing as revealed by flow cytometry. Interestingly, STS treatment and serum starvation resulted in a similar number of MPs exposing PCI on their surface (~60–70% in both cell lines).

To activate platelets and to generate platelet-derived MPs, we used the thrombin receptor agonist TRAP-6 and the calcium ionophore ionomycin. We found an increase in the number of annexin V positive MPs (2-fold), when washed platelets were treated with ionomycin, while TRAP-6, a more physiological stimulus for platelet activation, did not result in a higher number of MPs as compared to unstimulated platelets. The number of annexin V positive MPs obtained after platelet activation was therefore stimulus-dependent. Furthermore, the presence of PCI during platelet activation did not influence the amount of released MPs. PCI is stored in the α-granules of resting platelets. Upon platelet activation up to 30% of PCI is released and subsequently detected on the surface of platelets [42]. Nishioka et al. provided the first indication that activated platelets also release small sized particles, recognized as phospholipid membrane-derived microvesicles with surface-associated PCI. Our results further indicate that PCI-containing MPs are generated from activated platelets, since they also exposed the platelet activation marker CD62P on their surface. By determining the number of MPs exposing PCI, PS and CD62P, we found again a stimulus-dependence concerning the binding of PCI to the surface of MPs. MP generation by the platelet agonist TRAP-6 resulted in the same number of PCI positive, PS positive and CD62P positive MPs as compared to unstimulated platelets, but these TRAP-6 generated MPs were able to bind exogenous pPCI. In contrast, platelet activation with ionomycin resulted in a higher number of MPs exposing PCI, PS and CD62P, but additional binding of exogenous pPCI was not observed. These results suggest that possible binding sites for PCI such as PE and PS were already occupied and that saturation with PCI already occurred during the process of MP generation, when ionomycin triggered MP release.

Having shown that PCI is present in/on MPs generated *in vitro*, we aimed to study the occurrence of PCI on MPs present in human plasma from healthy individuals. Our results provide first evidence that these MPs contain PCI. MP-associated PCI is at least partially surface-exposed as revealed by ELISA and by flow cytometry. Moreover, these MPs carry PS as judged from annexin V binding and originate mainly from platelets/megakaryocytes as judged from anti-CD41-IgG binding. Circulating MPs from healthy individuals are most likely not derived from activated platelets since they do not expose CD62P on their surface. Considering the rapid MP clearance from the circulation, MPs would need to be continuously shed by a mechanism other than activation of mature platelets to account for the number of circulating CD41 positive/CD62P negative MPs in healthy individuals [58]. Our results therefore confirm previous data [13, 59] and strongly suggest that the majority of MPs in the plasma of healthy individuals is derived from megakaryocytes and not from activated platelets.

In previous studies we have shown that annexin V and PCI were co-localized in atherosclerotic plaques [41]. Furthermore, binding of PCI and annexin V occurred at the same sites on apoptotic Jurkat cells and U937 cells [45]. The presence of PCI on annexin V binding MPs is therefore an additional indication that PCI and PS interact *in vivo*. In the current study we did not observe additional binding of fluorophore labeled pPCI (pPCI-Cy3) to the surface of plasma-derived MPs, even when using very high concentrations of pPCI (500 nM). Plasma-derived MPs therefore seem to be saturated with PCI, which would furthermore suggest that saturation already occurs at a physiological PCI concentration (100 nM).

Binding of PCI to phospholipids can be competed by heparin [41]. In the present study we can show that MP-bound PCI was released by heparin in a concentration- and time-dependent manner, supporting the hypothesis that PCI is partially bound to phospholipids on MPs. It is therefore surprising that we were not able to compete already bound PCI with pPCI-Cy3. However, we cannot exclude that Cy-3 labeled PCI has a lower affinity for the PCI-binding sites on plasma-derived MPs as compared to native PCI. Also the stimulus that causes MP formation seems to influence binding of exogenously added PCI or pPCI-Cy3 (Fig 1D and S2 Fig, respectively). At the present stage, however, we could only speculate, why MPs isolated from the plasma of healthy donors behave differently with respect to binding of exogenously added PCI as compared to MPs generated *in vitro*.

MPs present a surface rich in phospholipids [6, 60]. We have shown before that certain phospholipids stimulate the activity of PCI towards thrombin and aPC [41]. Surprisingly, we could not observe any activity of MP-bound PCI, although most of the PCI detected on the surface of MPs corresponded to full length PCI (57 kDa). These results suggest that the RCL of PCI is not available for the interaction with its target proteases. Since PCI is synthesized by and stored in megakaryocytes, it might be that during MP formation from megakaryocytes, PCI is incorporated in the lipid bilayer of MPs in such a way that finally any interaction of the RCL of PCI with its target proteases is prevented. Previous results by our group have shown that PE selectively mediates membrane insertion and cell entry of PCI [44]. Indeed, another possibility is the binding of plasma PCI and its partial insertion into the lipid bilayer after MP release, which would also limit the conformational freedom of the originally very flexible RCL of PCI. Alternatively, PCI may be bound to other molecules (e.g. proteins) on MPs, which interfere with its inhibitory activity towards aPC and thrombin.

Even in the presence of heparin we did not observe any activity of MP-bound PCI. This is surprising, since heparin, which releases PCI from MPs (Fig 3) should be able to stimulate PCI activity in solution. However, other heparin binding proteins present on MPs such as vitronectin (Table 1) may interfere with the stimulatory effect of heparin. In fact, we have shown before that in the presence of heparin vitronectin protects urokinase from inhibition by PCI [61]. This may also be the case for the interaction of PCI with aPC and thrombin.

We have shown before using suspensions of pure phospholipids that PS, oxidized PE, cardiolipin, and some phosphoinositides stimulated PCI activity [41, 43]. We therefore studied whether MPs have an effect on the activity of exogenous rhPCI. However, we observed neither a stimulatory nor an interfering effect of MPs on aPC or thrombin inhibition by rhPCI. Nishioka et al. previously demonstrated the presence of PCI on platelet-derived microvesicles [42] and also showed that the activity of PCI towards aPC was stimulated by phospholipid vesicles. This effect was strongly dependent on PE since the inhibition of aPC by PCI increased in proportion to the amount of PE in phospholipid vesicles. The inhibition of aPC by PCI was only slightly enhanced in the presence of phospholipid vesicles containing 20% PE, whereas the inhibition of aPC by PCI increased up to 7-fold when phospholipids vesicles were containing 40% PE [42]. However, so far there is no data on the stimulation of PCI activity by platelet-derived microparticles. Further studies are needed to determine the conditions, under which phospholipids/phospholipid membranes can modulate PCI activity.

Co-precipitation of PCI with the complement components C1q, C3, C4a, and C9 provides the first indication that PCI might interact with microparticle-/phospholipid associated complement factors. Although extensive further studies are needed to confirm this hypothesis, our findings—together with our previous data on the effect of PCI on the phagocytosis of bacteria [44] and apoptotic cells [45]—suggest that PCI might have hitherto unrecognized immune-modulatory functions.

## Supporting Information

S1 Fig Induction of apoptosis in Jurkat cells. For the induction of apoptosis, Jurkat cells were either serum starved (control) or treated with STS (1 μM) for 3, 5.5 and 18 hours at 37°C. Afterwards, cells were harvested and stained with annexin V-EGFP and PI and subjected to flow cytometry. (A) Annexin V positive / PI negative cells (early apoptotic cells) and (B) annexin V positive / PI positive cells (late apoptotic cells) are depicted after serum starvation or STS treatment for different times as indicated. Data represent means and range of two independent experiments performed in duplicates.
(TIFF)

S2 Fig Binding of pPCI-Cy3 to MPs derived from Jurkat cells in the absence or presence of heparin. MPs were generated from Jurkat cells upon serum starvation (control) or treatment with STS (1 μM) for 18 hours at 37°C. Thereafter, MPs were isolated and incubated with pPCI-Cy3 (80 nM) in the absence or presence of heparin (10 U/ml) for 45 min at RT and analysed by flow cytometry. Data represent means and range of duplicates.
(TIFF)
